# Analysis of Acrylic and Methacrylic Networks through Pyrolysis-GC/MS

**DOI:** 10.3390/polym13244349

**Published:** 2021-12-12

**Authors:** Zakaria Belbakra, Alessandro Napoli, Zoubair Cherkaoui, Xavier Allonas

**Affiliations:** 1 Huntsman Advanced Materials, 4057 Basel, Switzerland; zakaria_belbakra@huntsman.com (Z.B.); alessandro_napoli@huntsman.com (A.N.); zoubair_m_cherkaoui@huntsman.com (Z.C.); 2 Laboratory of Macromolecular Photochemistry and Engineering, Université de Haute Alsace, 3b Rue Alfred Werner, 68093 Mulhouse, France

**Keywords:** pyrolysis-GC/MS, photopolymerization, acrylates, methacrylates

## Abstract

A direct analytical method developed to characterize UV-cured networks based on multi-step pyrolysis-gas chromatography-mass spectroscopy (GC/MS) is presented. Application of the method to characterize (meth)acrylate-based UV-cured networks is discussed. The reversion process of methacrylates is clearly observed during pyrolysis. In contrast, the decomposition of acrylates in high molecular weight degradation products is hardly detected. The potential impact of this technique to elucidate the structural and compositional nature of UV-cured polymeric networks is highlighted.

## 1. Introduction

Characterization at the molecular level of crosslinked polymers remains challenging with traditional analytical methods. Their intrinsic insolubility makes analysis by liquid chromatographic methods not feasible. Studies on the monomer conversion of UV-cured polymers have been conducted by monitoring the decay of reactive groups using spectrometric techniques like Fourier-transform infraRed spectroscopy (FT-IR), near infrared spectroscopy (NIR), and (high resolution) nuclear magnetic resonance (NMR) and by (photo)-differential scanning calorimetry (DSC) [1,2,3,4,5]. UV-cured network structures have been characterized using solid-state NMR [6,7]. In-depth measurement of conversion has been performed by confocal Raman microscopy [8,9]. In addition, dynamic mechanical analysis and swelling experiments have been conducted to provide crosslinking density data of crosslinked polymers [10,11,12]. More recently, matrix assisted laser desorption ionization—time of flight (MALDI-TOF) MS combined with supercritical methanolysis was used to characterize the crosslinking structures of UV-cured polymers [13,14,15]. Such indirect characterization methods can suffer from the lack of detailed compositional and structural information and/or can be time-consuming when working with industrial-grade products.

Pyrolysis-GC/MS has imposed itself as a powerful analysis tool for the chemical structure characterization of intractable materials. It found applications in areas as soil chemistry, natural and synthetic organic polymers (as vulcanized rubbers, polystyrene gels, epoxy resins and complex polymer systems, etc.) [16,17,18,19]. Several articles related to the characterization by Py-GC/MS of polymeric networks are available [20,21,22,23,24]. However, very few articles deal with the characterization of UV-cured polymers using Py/GC-MS. According to the literature, the main method used to characterize UV-cured materials was based on a derivatization method called “reactive pyrolysis”, or “thermochemolysis” or again “pyrolysis-alkylation-GC/MS”. Reactive pyrolysis-GC/MS was employed for the characterization of UV-cured acrylate resins, such as for the compositional analysis of multi-component acrylate resins [25], for the characterization of network structures in UV-cured poly (ethylene glycol) diacrylate (PEDA) [26], for the evaluation of the molecular weight of bisphenol-A type epoxy acrylate (BEA) prepolymer in UV-cured resins [27] and for the determination of the conversion of UV-cured pentaerythritol triacrylate (PETA) [28]. Although the method used in these studies is able to provide qualitative as well as quantitative results on crosslinked networks, the reactive Py-GC/MS method was revealed to be time-consuming and can reveal some complexity in experiment implementation and data interpretation due to its indirect character. These latter points motivated us to develop a direct and rapid method enabling us to catch useful information on UV-cured networks.

In this paper, a direct analytical method developed to characterize UV-cured networks based on multi-step pyrolysis/GC-MS is presented. Application of the method to characterize (meth) acrylate-based UV-cured networks is discussed. The potential impact of this technique to elucidate the structural and compositional nature of UV-cured polymeric networks is highlighted.

## 2. Materials and Methods

Two formulations were studied based on commercially available photocurable resins (a) difunctional ethoxylated-3-bisphenol-A-dimethacrylate EBADMA (SR 348c from Sartomer, Paris, France, Mw = 496 g·mol^−1^) and (b) difunctional ethoxylated-3-bisphenol-A-diacrylate EBADA (SR 349 from Sartomer, Mw = 424 g·mol^−1^). The photoinitiator was 2,4,6-trimethylbenzoyldiphenylphosphine oxide (Lucirin TPO from BASF, Ludwigshaffen, Germany, Mw = 348 g·mol^−1^) at 2 wt%. The curing process was performed first by coating a thin film over a glass substrate using a 24 µm automatic bar coater. Then, the coating was irradiated under nitrogen gas flow with UVA at 5 J/cm^2^ using a BS04 irradiation chamber.

Pyrolysis samples were prepared by introducing 10 to 100 µg of resin in a quartz tube containing glass wool.

The pyrolyzer used is a Programmable CDS 5200 vertical pyrolyzer built by CDS analytical (Oxford, PA, USA). The latter comprises a heating probe equipped with a platinum filament which can be heated up to 1400 °C at a heating rate of 10 °C to 20,000 °C/s. This pyrolyzer allows multi-step temperature pyrolysis with the use or not of a reactant gas via the CDS5000 Pyroprobe software. In addition, this pyrolyzer is equipped with a cryotrap permitting it to trap volatiles before being injected. The GC-MS equipment is constituted of a CP-3800 gas chromatograph (Varian, Walnut Creek, CA, USA) equipped with a high temperature Zebron ZB-1HT capillary column (−60 °C to 400–430 °C, Phenomenex, Torrance, CA, USA) coated with 100% dimethyl polysiloxane stationary phase. The column dimensions are 30 m length × 0.25 mm internal diameter with 0.10 µm film thickness. The GC is coupled with a triple quadrupole MS 300 mass spectrometer from Varian.

Thermogravimetric analysis (TGA) experiments were carried out using a Mettler TG50 thermogravimeter. TGA were performed from 25 °C to 800 °C at 20 °C/min and under a nitrogen flow of 20 mL/min.

Pyrolyzer, GC, and MS instruments were set in order to obtain good performance in terms of volatiles transfer from pyrolyzer to GC and from GC to the mass spectrometer and in terms of chromatograms reproducibility (signal intensities, retention time) and resolution (peaks separation).

Several experiments were realized in order to find the most suitable analysis time. First experiments were realized with a total analysis time of about 130 min. One analysis comprises a system cleaning, a blank before analysis, and the three degassing steps of the degassing stage followed by the pyrolysis steps. Furthermore, before each step, an equilibration time was needed (30 s). The parameter which could really influence the analysis time was the temperature gradient of the GC. The first settings of the temperature program lead to a chromatograph analysis time lasting 32 min. By decreasing the chromatograph analysis time to 20 min and by removing the cleaning step, the total analysis time was decreased to 80 min. However, these changes should not involve quality data losses. To check if this analysis time reduction is feasible, a comparison between the direct Py-GC/MS with the different analysis time was performed over a di(meth)acrylate resin was repeated with the new GC parameters. It was observed that the chromatograms resulting from the two different analysis times showed the same responses in terms of peaks numbers and intensity of the peaks. Moreover, it was observed that the shorter analysis time enabled the observation of a higher molecular weight monomer that was not observed in the chromatogram resulting from the longer analysis time. The other parameters are given in Table A1 in Appendix A.

## 3. Results

### 3.1. Prerequisites

**Scheme 1:**
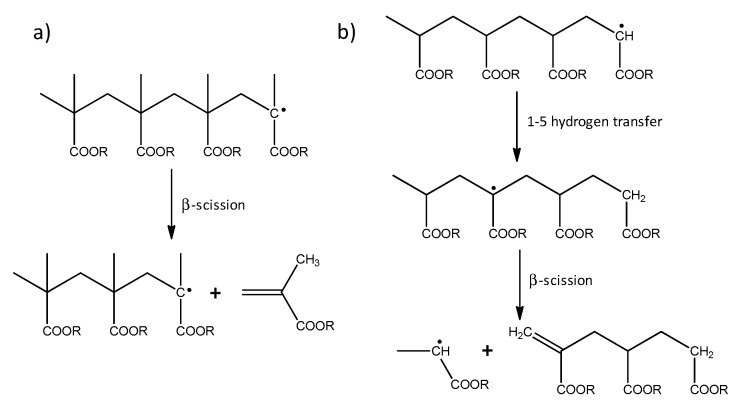
β-scission occurring in methacrylate polymer chains (**a**) and acrylate polymer chains (**b**).

The pyrolysis of poly(methyl methacrylate) (PMMA) is a typical example of the degradation mechanism of methacrylates. The PMMA pyrolysis results in depolymerization, giving mainly methyl methacrylate monomers. Under the heat, a C-C scission takes place, leading to a tertiary carbon-centered radical. It was shown that the degradation mechanism involves β-scissions over the methacrylate polymer chain, as depicted in Scheme 1. It was also shown that the R group does not affect the monomer reversion process and that the process is unchanged for copolymers. Ref. [29] This could be understood by analyzing the methacrylate structure. Indeed, it can be seen that the C-C bonds from the methacrylate chain are the weakest and produce the most stable free radicals. The free radicals produced by breaking the C-C bonds result in tertiary stabilized free radicals, which are the most stable radicals. Moreover, there is no possibility of hydrogen transfer which could transfer the free radical elsewhere in the chain. The breaking of the C-CH_3_ bonds should lead to a less stabilized free radical. Therefore, no other pathway than the C-C bonds breaking via beta scission can be envisaged for methacrylate polymers considering free radical stability preferences. After the formation of the tertiary radical, a monomer reversion process takes place (called also unzipping process), leading to methacrylate monomers as the most stable fragments. However, it should be noted that side reactions may occur during degradation, such as radical–radical recombination or intermolecular chain transfer.

In acrylate-based polymers, hydrogens are present instead of the methyl groups in the polymer chain. That let the possibility for intramolecular hydrogen transfer reaction. The cleavage of the C-C bond from the polymer chain leads to a free radical on a secondary carbon which is then stabilized by hydrogen transfer from carbon 1 to carbon 5, thereby creating a free radical stabilized on a tertiary carbon. After the formation of the tertiary radical, a β-scission occurs, resulting in the formation of a stable acrylate trimer as depicted in Scheme 1 [29,30].

### 3.2. Degassing Temperature Determination

Before analyzing the uncured and cured samples, the degassing temperature has to be determined in order to define the best direct multi-step Py-GC/MS method. Then, the uncured EBADMA resin was analyzed by first identifying the main chromatographic peaks. The analysis of the cured EBADMA resin was performed in the same way.

**Figure 1:**
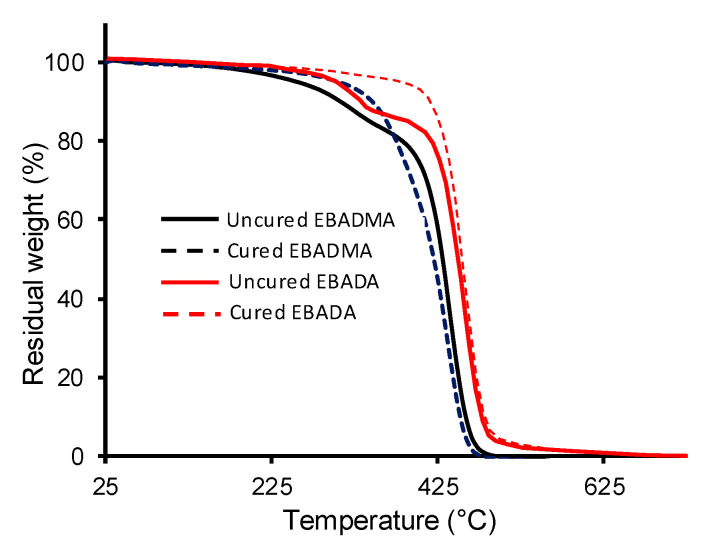
Thermograms of EBADMA and EBADA uncured and cured samples.

First, TGA were performed on the uncured and cured resins to determine the temperatures corresponding to the onset of their degradation. TGA thermograms obtained for the uncured and cured methacrylic and acrylic resins showed an onset temperature before degradation of around 325 °C (Figure 1).

**Figure A1:**
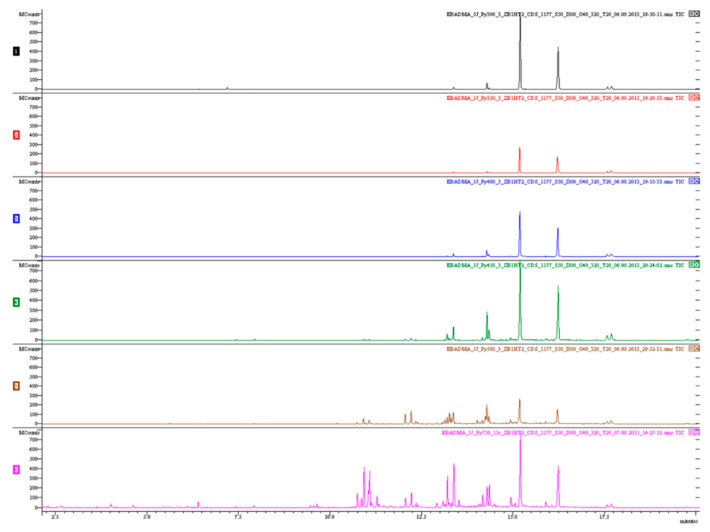
Cured EBADMA chromatograms obtained using multi-step Py-GC/MS degassing temperature determination method.

**Figure A2:**
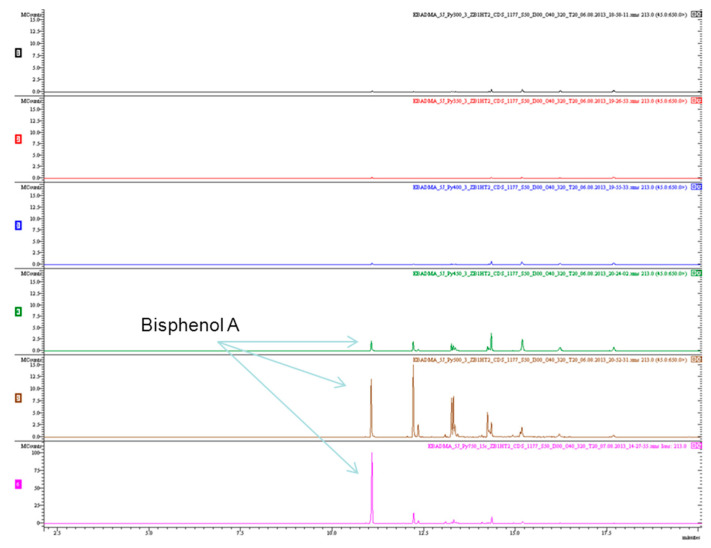
Cured EBADMA chromatograms at different degassing temperatures: cured EBADMA degradation monitoring using bisphenol-A base peak of *m*/*z* = 213.

According to these results, it was decided that the degassing zone to the screen using multi-step Py-GC/MS would be 300 °C to 500 °C using an increment of 50 °C. The cured sample was heated for 3 min at each temperature before gas chromatogram acquisition. Then, a pyrolysis step at 750 °C during 15 s was incorporated to help to identify the degradation products present in the pyrolysis chromatogram. From the different chromatograms obtained at different successive temperatures for a cured EBADMA sample, it is possible to control the degradation by monitoring the chromatographic peak of bisphenol A base peak which has an *m*/*z* = 213 (Figure A1). The bisphenol-A chromatographic peak should increase with degradation advancement. If we filter the chromatograms using ion *m*/*z* = 213, the chromatograms clearly show that the bisphenol A degradation is increasing with temperature (Figure A2). According to these chromatograms, the degassing temperature was chosen to be 400 °C for the cured EBADMA resin. Therefore, the direct multi-step Py-GC/MS method, which will be used to analyze the uncured and cured resin, consists of three degassing steps at 180 °C of 180 s, 180 s, and 60 s followed by a pyrolysis step at 750 °C during 15 s. Similar results are obtained for EBADA, although that the degradation occurs at a slightly higher temperature. Accordingly, the degassing temperature will be fixed at 400 °C.

### 3.3. Analysis of Uncured EBADMA Resin

**Figure A3:**
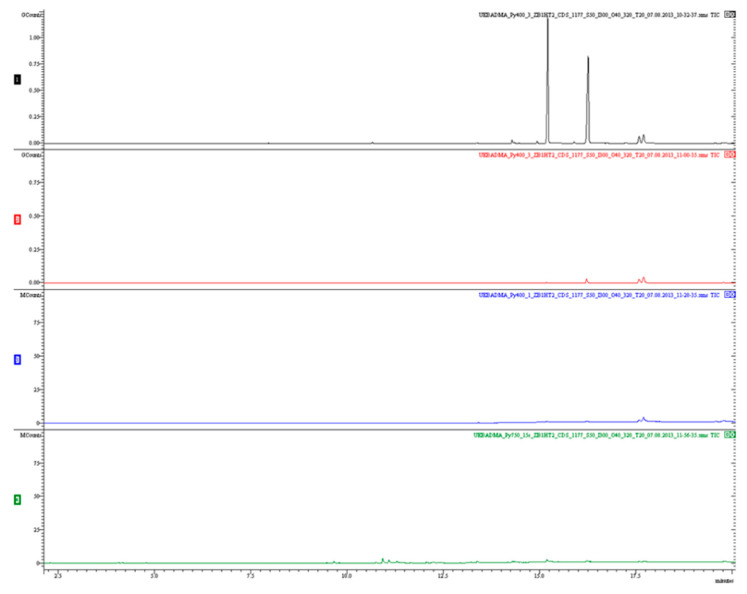
Chromatograms obtained for an uncured EBADMA sample.

When applying this methodology to uncured EBADMA almost all volatiles came out during the two first steps (Figure A3). Small amounts of volatiles came out at the third degassing step, and a negligible amount of pyrolysis products came out during the pyrolysis step. It is worth noting that the quartz tube was empty after the experiment, and no char formation was observed on the quartz tube walls.

**Scheme A1:**
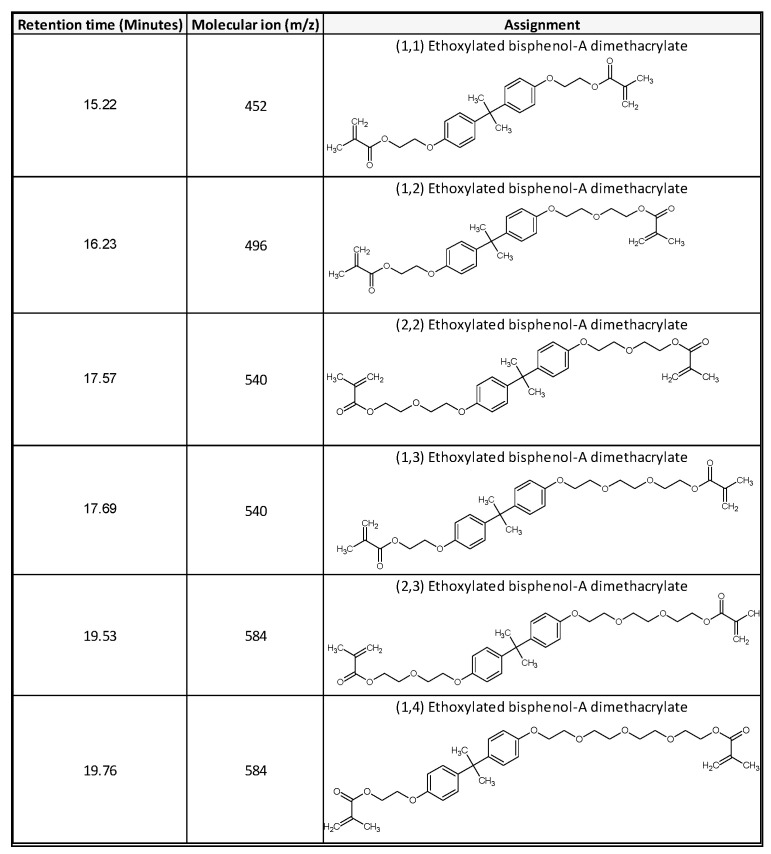
Peaks assignment of the main peaks of the chromatogram obtained from the first degassing step of the uncured EBADMA resin.

**Figure A4:**
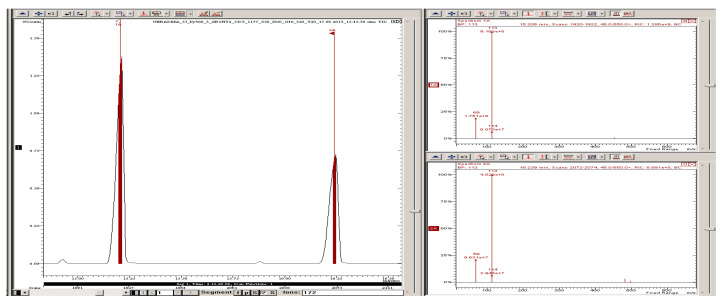
Chromatographic peaks of the 2× ethoxylated bisphenol-A dimethacrylate and the 3× ethoxylated bisphenol-A dimethacrylate and their associated mass spectra.

**Figure A5:**
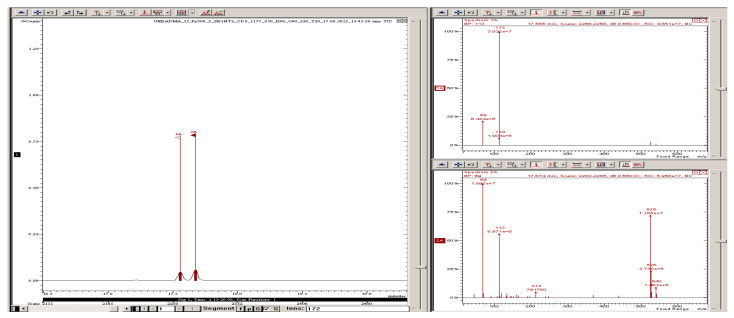
Chromatographic peaks of the 4× ethoxylated bisphenol-A dimethacrylates and their associated mass spectra.

**Figure A6:**
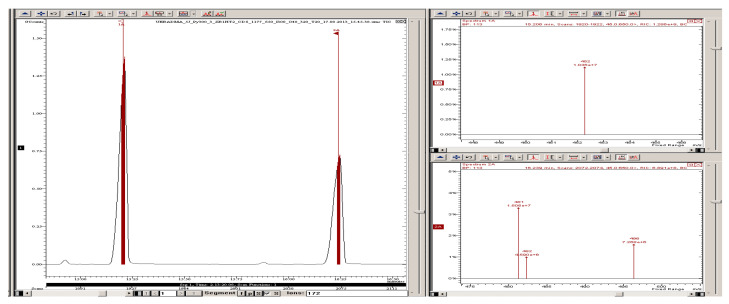
Zoom of mass spectra in the molecular ion region of the chromatographic peaks of the 2× ethoxylated bisphenol-A dimethacrylate and the 3× ethoxylated bisphenol-A dimethacrylate.

**Figure A7:**
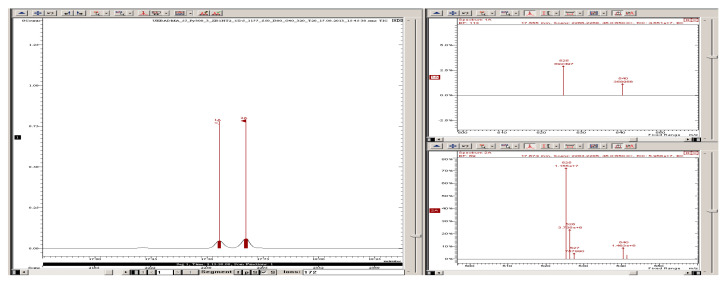
Zoom of mass spectra in the molecular ion region of the two chromatographic peaks of the 4× ethoxylated bisphenol-A dimethacrylates.

**Figure A8:**
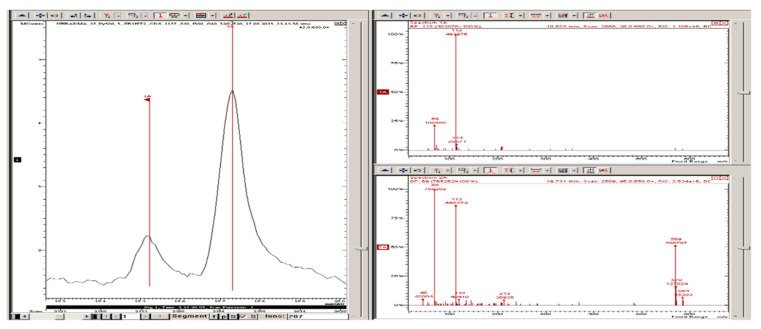
Chromatographic peaks of the 5× ethoxylated bisphenol-A dimethacrylates and their associated mass spectra.

**Figure A9:**
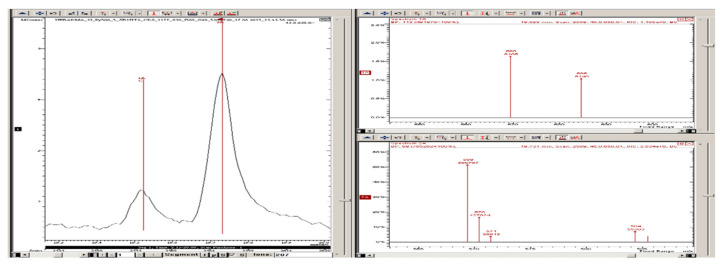
Zoom of mass spectra in the molecular ion region of the two chromatographic peaks of the 5× ethoxylated bisphenol-A dimethacrylates.

Six main peaks exhibiting different retention times were identified as several ethoxylated bisphenol-A dimethacrylate monomers using mass spectra analysis (Table 1 and Scheme A1 for structures). All the mass spectra corresponding to these peaks show characteristic fragments of the bisphenol-A dimethacrylate resin (Figure A4 and Figure A5). In all the spectra, the base peak having an *m*/*z* = 113 corresponding to the ethyl methacrylate fragment, as well as the methacryloyl peak having an *m*/*z* = 69 are present. Furthermore, if zoom is done in the molecular ion region, it is possible to recognize the molecular ions of the different major monomers present in the resin (Figure A6 and Figure A7). From these results, it is possible to identify 2× ethoxylated bisphenol-A dimethacrylate (*m*/*z* = 452) at a retention time RT = 15.22 min and 3× ethoxylated bisphenol-A dimethacrylate (*m*/*z* = 496) at RT = 16.23 min. The fragment having an *m*/*z* = 481 corresponds to the molecular ion minus 15 typically coming from the methyl group of the bisphenol A molecule. Two other peaks could be attributed to 4× ethoxylated bisphenol-A dimethacrylate, this molecule being present in two configurations: the (2,2) ethoxylation of the bisphenol-A at RT = 17.57 min and the (1,3) ethoxylation of the bisphenol A at RT = 17.69 min. The mass spectra of these two peaks confirm this by showing that both spectra have the same molecular ion of *m*/*z* = 540. Finally, two very small peaks appearing at the end of the chromatogram at RT= 19.53 min and RT = 19.73 min were attributed to the 5× ethoxylated bisphenol-A dimethacrylates (Figure A8 and Figure A9). Two peaks were also observed for this molecule since the five ethoxylation can be present in two configurations: the (1,4) ethoxylation of the bisphenol-A and the (2,3) ethoxylation of the bisphenol A. The mass spectra of these two last peaks show that both spectra have the same molecular ion of *m*/*z* = 584, confirming that the two peaks correspond to the 5× ethoxylated bisphenol-A dimethacrylate. The latter will be not taken into account in the chromatographic data interpretation since the corresponding peaks are too small.

Table 1 reports the peaks areas in counts/s of the four main peaks at the different steps. It can be seen that there are more 2× ethoxylated bisphenol-A dimethacrylate monomers (about 50.89%) than 3× ethoxylated bisphenol-A dimethacrylate monomers (41.72%). The amount of 4× ethoxylated bisphenol-A dimethacrylate represents only about 7.4% of the total monomers. It is important to underline that these types of information cannot be obtained with common characterization techniques.

### 3.4. Analysis of 5 J/cm^2^ Cured EBADMA Resin

**Figure 2:**
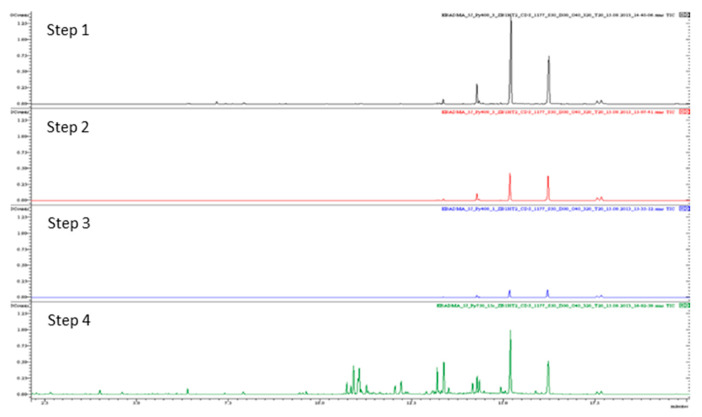
Chromatograms obtained for a 5 J/cm^2^ cured EBADMA sample.

The multistep direct Py-GC/MS analysis of the cured EBADMA resin was performed in the same conditions as for the previous uncured resin. Figure 2 shows the chromatograms obtained for a cured EBADMA sample. It can be seen that the majority of the monomers are going out during the two first degassing steps; there are just a few remaining monomers coming out in the third degassing step. In addition, the chromatogram corresponding to the pyrolysis step contains several peaks with high intensities showing the degradation of the cured EBADMA network, which remained after the three degassing steps.

Table 2 reports the peaks areas of the four major monomers at the different steps and reports also the sums of peaks areas for each monomer through the three degassing steps. The sums of peaks areas were then normalized by the effective mass of the resin (93.1 µg), which corresponds to 98% of the pyrolyzed mass (95 ± 1 µg) since 2% corresponds to the photoinitiator mass. The overall degassed monomers content $Sf_{1}$ in counts/s/µg was obtained by the addition of the normalized sums of peaks areas obtained for each degassed monomers. In addition, the table reports the distribution of the degassed monomer in the resin. It is interesting to see that the monomers are going out from the cured resin in relatively the same proportion as in the uncured resin.

Values obtained from the multi-step direct Py-GC/MS analysis of the uncured and the cured EBADMA resin can be used to calculate the average conversions of the individual monomers and the average conversion of the overall monomers in the cured resin. By comparing the normalized content of degassed monomers of uncured EDABMA to that of the cured one, an overall monomers conversion of 70.9% was found for the 5 J/cm^2^ cured EBADMA resin. Table 2 reports the individual average monomer conversions for the different ethoxylated bisphenol-A dimethacrylate monomers present in the resin. It can be seen here also that the four different monomers have almost the same conversion (around 70%) with a little bit higher conversion for the 3× ethoxylated bisphenol-A dimethacrylate (−73%). Those types of results can give important information about monomer reactivities. Here, for instance, it can be seen that these monomers have almost the same reactivity and that the network is composed on average of the four monomers.

**Figure 3:**
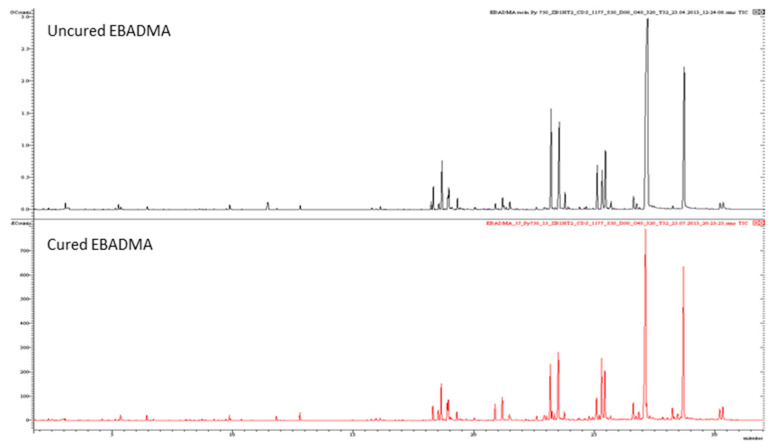
Comparison of the Py-GC/MS chromatograms obtained from the uncured (**top**) and the cured (**bottom**) EBADMA resin pyrolyzed at 750 °C during 15 s.

It was seen that the analysis of Py-GC/MS results could be used to obtain interesting information on the network structure composition. In order to try to get some information regarding the crosslinked network structure, Py-GC/MS chromatograms obtained from pyrolysis at 750 °C during 15 s of the uncured resin and the cured resin were compared. Figure 3 shows that the two chromatograms coming from the pyrolysis of the uncured and the cured EBADMA resin do not show any remarkable differences, i.e., there are no specific pyrolysis fragments of the cured resin. It seems that the degradation scheme of the cured resin is the same as that of the uncured one. That is due to the monomer reversion process that undergoes cured methacrylate resins. Ref.[29] The cured resin is degrading by reforming the initial monomers. This explains the fact that no other specific fragments were identified for the methacrylate resins.

### 3.5. EBADA Uncured Resin Analysis

**Figure A10:**
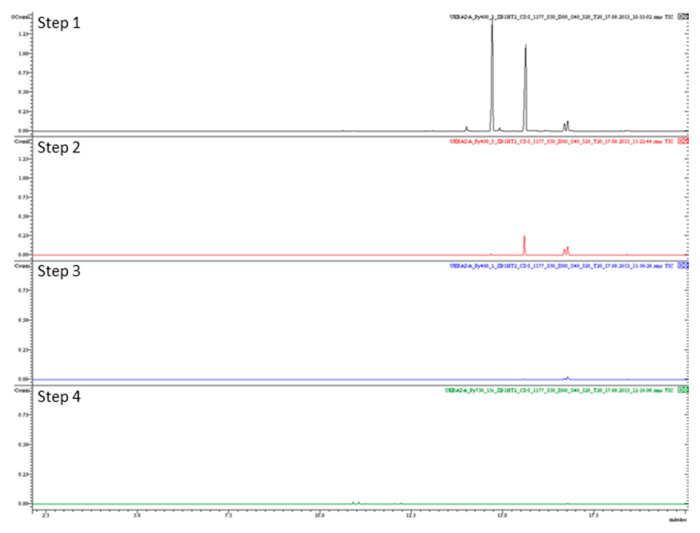
Chromatograms obtained for an uncured EBADA sample.

Using a degassing temperature of 400 °C, the direct multi-step Py-GC/MS method was applied to both uncured and cured EBADA. As for the previous methacrylate, it can be seen that almost all volatiles came out during the two first steps (Figure A10). Very small amounts of volatiles came out at the third degassing step. In addition, a very small amount of pyrolysis products came out during the pyrolysis step of the uncured EBADA resin showing that almost all the volatiles came out during the degassing steps. The fact that the quartz tube after the experiments was empty and no char formation was observed in the quartz tube walls comfort the assumption that all the volatiles came out.

**Scheme A2:**
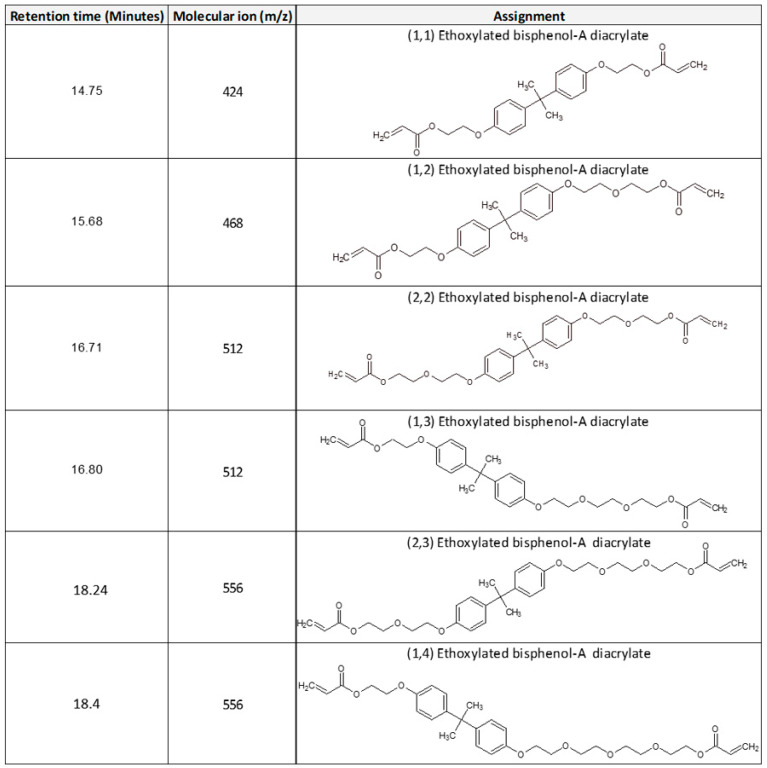
Peaks assignment of the main peaks of the chromatogram obtained from the first degassing step of the uncured EBADA resin.

**Figure A11:**
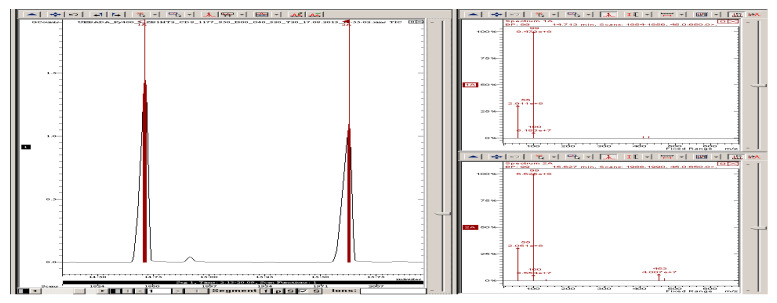
Chromatographic peaks of the 2× ethoxylated bisphenol-A diacrylate and the 3× ethoxylated bisphenol-A diacrylate and their associated mass spectra.

**Figure A12:**
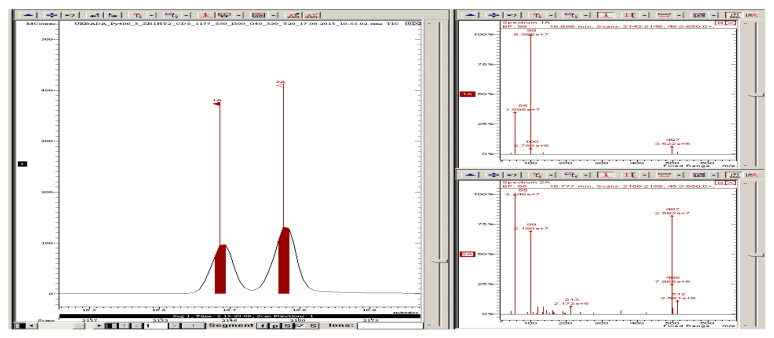
Chromatographic peaks of the 4× ethoxylated bisphenol-A diacrylates and their associated mass spectra.

The main peaks related to the monomers were identified using mass spectra analysis. The same trend observed for the EBADMA is observed for the EBADA resin. It seems that there are several ethoxylated bisphenol-A diacrylate monomers in the resin (Table 3 and Scheme A2 for the structures). Mass spectra (Figure A11 and Figure A12) show characteristic fragments of the bisphenol-A diacrylate resin. In all the spectra, the base peak (*m*/*z* = 99) corresponding to the ethyl acrylate fragment as well as the acryloyl peak (*m*/*z* = 55) are present. It is also possible to recognize the molecular ions of the different major monomers present in the resin. Thus, the first main peak was attributed to the 2× ethoxylated bisphenol-A diacrylate since its corresponding mass spectrum showed a molecular ion of *m*/*z* = 424. The ion *m*/*z* = 409 corresponds to the molecular ion minus 15 coming from the removing of a methyl group in the bisphenol moiety. Then, the second peak was attributed to the 3× ethoxylated bisphenol-A diacrylate since its corresponding mass spectrum showed the molecular ion of *m*/*z* = 468. Finally, the third and the fourth main peaks were attributed to the 4× ethoxylated bisphenol-A diacrylate. Indeed, as seen for the 4× ethoxylated bisphenol-A dimethacrylate, two peaks were observed for the latter molecule since the four ethoxylation can be present in two configurations: the (2,2) ethoxylation of the bisphenol-A or the (1,3) ethoxylation of the bisphenol A. This is confirmed by the mass spectra of these two peaks, showing that both spectra have the same molecular ion of *m*/*z* = 512. As for EBADMA, the 5× ethoxylated bisphenol-A diacrylate having an *m*/*z* = 556 was identified. The chromatogram shows the two chromatographic peaks of the 5× ethoxylated bisphenol-A diacrylate with their respective mass spectra. In addition, two peaks were observed for the latter molecule since the five ethoxylations can be present in two configurations: the (1,4) ethoxylation of the bisphenol-A and the (2,3) ethoxylation of the bisphenol A. Peaks corresponding to the ethyl acrylate ion fragment of *m*/*z* = 99 and to the acryloyl ion fragment of *m*/*z* = 55 are predominantly present in the mass spectra of the two chromatographic peaks. The mass spectra of these two last peaks show that both spectra have the same molecular ion of *m*/*z* = 556, confirming that the two peaks correspond to the 5× ethoxylated bisphenol-A diacrylate. The latter will not be taken into account in the chromatographic data interpretation since the corresponding peaks are too small. Table 3 reports the main peak assignments.

Minor additional peaks identified as being ethoxylated bisphenol-A monoacrylates were found. These compounds exhibit mass spectra that contain the ethyl acrylate base peak *m*/*z* = 99 and the acryloyl peak at *m*/*z* = 55. The first peak at about RT = 14.01 min has a molecular ion of *m*/*z* = 370, which could correspond to the (0,2) Ethoxylated bisphenol-A monoacrylate. The other peak at RT = 14.91 min has a molecular ion of *m*/*z* = 414, which could correspond to the (0,3) Ethoxylated bisphenol-A monoacrylate.

### 3.6. Analysis of 5 J/cm^2^ Cured EBADA Resin

**Figure 4:**
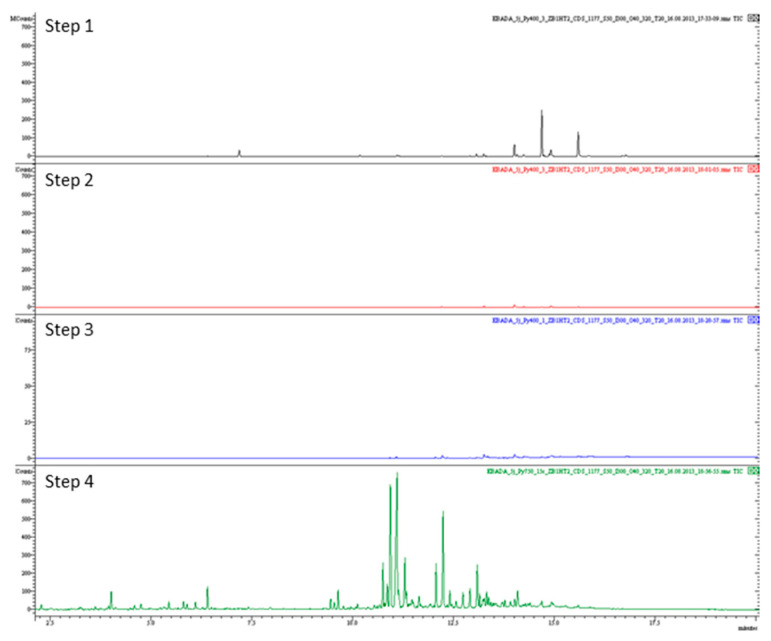
Chromatograms were obtained for a 5 J/cm^2^ cured EBADA sample.

The multi-step direct Py-GC/MS analysis of the cured EBADA resin was performed in the same way as for the previous uncured resin. Figure 4 represents the chromatograms obtained for a cured EBADA sample.

It can be seen that the majority of the monomers are going out during the first degassing steps and no degradation fragments appeared before the pyrolysis step. The chromatogram corresponding to the pyrolysis step contains several peaks with high intensities showing the degradation of the cured EBADA network, which remained after the degassing steps.

Table 4 reports the peaks areas of the four degassed monomers at the different steps and reports also the sums of peaks areas for each monomer through the degassing steps. It seems that the monomers are going out from the cured resin in different proportions than in the uncured resin. Around 59% of 2× Ethoxylated bisphenol-A diacrylate is degassing from the cured resin, whereas around 36% only of 3× ethoxylated bisphenol-A diacrylate is degassing from the cured resin. In addition, the 5.2% only of the 4× ethoxylated bisphenol-A diacrylates are degassing from the cured resin.

As for the EBADMA resin experiment, values obtained from the multi-step direct Py-GC/MS analysis of the uncured and the cured EBADA resin were used to calculate the average conversions of the individual monomers constituting the resin and the average conversion of the overall monomers in the cured resin. An overall average monomer conversion of 97.29% was found for the 5 J/cm^2^ cured EBADMA resin. Table 4 reports the estimated monomer conversion average for the different ethoxylated bisphenol-A diacrylate monomers present in the resin. It can be seen that the four different monomers have a similar conversion (around 98%).

In order to try to get some information regarding the crosslinked network structure, Py-GC/MS chromatograms obtained from pyrolysis at 750 °C during 15 s of the uncured resin and the cured resin were compared. The two chromatograms coming from the pyrolysis of the uncured and the cured EBADA resin do not show any remarkable differences and exhibit almost the same fingerprint. Contrary to methacrylate-based monomers, the degradation of acrylate monomers does not follow a monomer reversion process. Indeed, no monomers peaks were identified in the chromatogram of the cured EBADA resin. However, no special network fragment was identified in the chromatogram. It is known that acrylates generally degrade following β-scission process and produce mainly trimers and dimers. Here, dimers and trimers molecular weights are so big that it will not be possible to detect them in the current Py-GC/MS configuration. A time-of-flight mass analyzer should be more adapted to detect high molecular weight fragments. Also, it was not yet possible to identify some crosslinking fragments because of the multitude of peaks present in the pyrolysis chromatograms. Simpler UV-cured systems should help for the identification of these fragments.

In the same way as the EBADMA analyses, the multi-step direct Py-GC/MS method developed enabled first to identify different EBADA monomers presenting different ethoxylation numbers in a resin supposed to contain just the 3× ethoxylated bisphenol-A diacrylate monomer. Moreover, the monomer compositions could be drawn. Secondly, a conversion average of 97.29% of the different monomers was calculated. Also, conversions averages of the individual monomers were calculated, showing similar conversions values (about 98%) for the different monomers. Then, the quantification of the EBADA cured resin was performed, showing the potential of the developed method. Finally, it was seen that the pyrolysis of the diacrylate networks led to several pyrolysis fragments difficult to identify. Also, it seems that the degradation behavior of the cured and the uncured are similar since the pyrolysis chromatograms patterns were similar.

## 4. Conclusions

A simple and fast method based on multi-step direct pyrolysis-GC/MS enabling the characterization of UV cured (meth)acrylate networks was presented. The reliability of the technique was successfully demonstrated through the analysis of uncured and cured formulated (meth)acrylate resins. The quality of the results was demonstrated through the fairly good reproducibility obtained without the use of any additional internal reference. By monomer structure analysis, it was shown that the composition of commercially available products may differ from the ideal single-molecule description commonly used. The distributions, the individual and overall monomers conversions were calculated through the monomers degassing contents.

However, the characterization of the network structure was still difficult. Indeed, pyrolysis of the cured methacrylate resin successfully showed that methacrylates undergo a monomer reversion process. The pyrolysis of the cured diacrylate resin did not enable the identification of specific network fragments. It would be interesting to study the copolymerization between acrylates and methacrylates. The pyrolysis of the cured resin was found similar to that of the uncured resin. According to the literature, the analysis toward the higher molecular weight fragments should give some information on the crosslinked network. The current mass spectrometer analyzer does not allow the analysis of high molecular weight fragments. The use of a TOF or ion cyclotron resonance analyzer instead of the triple quadrupole analyzer should permit the detection of higher ions fragments. The study of the polymer network at different conversions would afford important information on the building process of the network.

The use of high-purity low-molecular-weight monomers should permit fewer complex chromatograms and the extraction of information on network crosslinks. The application of the developed method to other polymeric materials such as epoxy resins and their curing agents is ongoing.

## Figures and Tables

**Table 1:** Peaks assignment of the chromatogram obtained from the first degassing step of the uncured EBADMA resin ^a^.

	(1,1) EBADMA	(1,2) EBADMA	(2,2) EBADMA	(1,3) EBADMA	(2,3) EBADMA	(1,4) EBADMA
Mw (g/mol)	452	496	540	540	584	584
RT (min)	15.22	16.23	17.57	17.69	19.53	19.76
Step 1 counts/s	8.22 × 10^9^	3.73 × 10^9^	8.57 × 10^7^	1.40 × 10^8^		
Step 2 counts/s	3.91 × 10^8^	2.86 × 10^9^	2.05 × 10^8^	2.92 × 10^8^		
Step 3 counts/s	1.06 × 10^7^	4.44 × 10^8^	1.45 × 10^8^	2.31 × 10^8^		
Step 4 counts/s	4.47 × 10^7^	7.11 × 10^7^	6.01 × 10^7^	1.00 × 10^8^		
Distribution (%)	50.9	41.7	2.9	4.5		

^a^ All experiments were repeated three times. Standard deviation of 5%.

**Table 2:** Py-GC/MS results for a 5 J/cm^2^ cured EBADMA sample ^a^.

	(1,1) EBADMA	(1,2) EBADMA	(2,2) EBADMA	(1,3) EBADMA
Step 1 counts	3.33 × 10^9^	1.98 × 10^9^	1.25 × 10^8^	1.62 × 10^8^
Step 2 counts	7.91 × 10^8^	8.60 × 10^9^	1.23 × 10^8^	1.70 × 10^8^
Step 3 counts	2.11 × 10^7^	2.56 × 10^8^	6.47 × 10^8^	1.04 × 10^8^
Step 4 counts	2.22 × 10^9^	1.15 × 10^9^	1.15 × 10^9^	1.15 × 10^9^
Distribution (%)	52.8	38.0	3.8	5.3
Conversion (%)	69.2	72.9	71.6	71.2

^a^ All experiments were repeated three times. Standard deviation of 10%.

**Table 3:** Peaks assignment of the chromatogram obtained from the first degassing step of the uncured EBADA resin ^a^.

	(1,1) EBADA	(1,2) EBADA	(2,2) EBADA	(1,3) EBADA	(2,3) EBADA	(1,4) EBADA
Mw (g/mol)	424	468	512	512	556	556
RT (min)	14.75	15.68	16.71	16.80	18.24	18.4
Step 1 counts	5.55 × 10^9^	3.50 × 10^9^	2.31 × 10^8^	3.25 × 10^8^		
Step 2 counts	5.20 × 10^7^	1.95 × 10^9^	2.49 × 10^8^	3.67 × 10^8^		
Step 3 counts	3.89 × 10^6^	1.47 × 10^8^	1.32 × 10^8^	2.34 × 10^8^		
Step 4 counts	9.32 × 10^6^	9.32 × 10^6^	2.60 × 10^7^	6.19 × 10^7^		
Distribution (%)	43.7	43.6	5.0	7.7		

^a^ All experiments were repeated three times. Standard deviation of 1%.

**Table 4:** Peaks assignment of the chromatogram obtained from the first degassing step of the cured EBADA resin ^a^.

	(1,1) EBADA	(1,2) EBADA	(2,2) EBADA	(1,3) EBADA
Step 1 counts	3.83 × 10^8^	2.32 × 10^8^	1.16 × 10^7^	1.84 × 10^7^
Step 2 counts	4.21 × 10^6^	4.67 × 10^6^	1.50 × 10^6^	2.83 × 10^6^
Step 3 counts	1.99 × 10^6^	0	0	0
Step 4 counts	0	0	0	0
Distribution (%)	58.9	35.9	2.0	3.2
Conversion (%)	96.1	97.7	99.1	99.1

^a^ All experiments were repeated three times. Standard deviation of 10%.

## Data Availability

The data presented in this study are available on request from the corresponding author. The data are not publicly available due to ongoing proprietary work but are available from the corresponding author on reasonable request.

## Appendix A

**Table A1:** Pyrolysis-GC/MS parameters used to perform pyrolysis experiments.

**Pyrolysis**	**parameters**
Helium purge flow rate	20 PSIG
Interface temperature in rest mode	60 °C
Interface temperature during pyrolysis	300 °C
Heating rate	20,000 °C/s
Volatiles collection time	4 min
Valve oven temperature	300 °C
Transfer line temperature	300 °C
**GC**	**parameters**
Oven temperature	40 °C
Volatiles concentration time to column	2 min
Injector temperature	300 °C
Split ratio	1/50
Gas carrier flow rate	Helium 20 PSIG
GC oven temperature program	(1) hold 1 min et 40 °C (2) 40 °C to 320 °C at 20 °C/min (3) hold 5 min at 320 °C
**MS triplet quadrupole**	**detector parameters**
Transfer line temperature	280 °C
Source temperature	260 °C
Source type	EI at 70 eV
*m*/*z* scan range	45 to 650
**Softwares**	
CDS 5000 Pyroprobe V. 4.02	
Varian MS Workstation V. 6.9	
NIST Database NIST MS search 2.0	
AMDIS V. 2.64	
